# Standing Air Bubble-Based Micro-Hydraulic Capacitors for Flow Stabilization in Syringe Pump-Driven Systems

**DOI:** 10.3390/mi11040396

**Published:** 2020-04-10

**Authors:** Yidi Zhou, Jixiao Liu, Junjia Yan, Tong Zhu, Shijie Guo, Songjing Li, Tiejun Li

**Affiliations:** 1 School of Mechanical Engineering, Hebei University of Technology, Tianjin 300132, China; 201811201007@stu.hebut.edu.cn (Y.Z.); 201831204033@stu.hebut.edu.cn (J.Y.); bc19920722@163.com (T.Z.); guoshijie@hebut.edu.cn (S.G.); 1993076@hebut.edu.cn (T.L.); 2 Hebei Key Laboratory of Robot Perception and Human-Robot Interaction, Hebei University of Technology, Tianjin 300401, China; 3 Department of Bioengineering, University of California, Berkeley, CA 94720, USA; 4 School of Mechatronics, Harbin Institute of Technology, Harbin 150001, China; lisongjing@hit.edu.cn

**Keywords:** fluidic capacitors, bubble-based, flow regulation, theoretical model, experimental studies

## Abstract

Unstable liquid flow in syringe pump-driven systems due to the low-speed vibration of the step motor is commonly observed as an unfavorable phenomenon, especially when the flow rate is relatively small. Upon the design of a convenient and cost-efficient microfluidic standing air bubble system, this paper studies the physical principles behind the flow stabilization phenomenon of the bubble-based hydraulic capacitors. A bubble-based hydraulic capacitor consists of three parts: tunable microfluidic standing air bubbles in specially designed crevices on the fluidic channel wall, a proximal pneumatic channel, and porous barriers between them. Micro-bubbles formed in the crevices during liquid flow and the volume of the bubble can be actively controlled by the pneumatic pressure changing in the proximal channel. When there is a flowrate fluctuation from the upstream, the flexible air-liquid interface would deform under the pressure variation, which is analogous to the capacitive charging/discharging process. The theoretical model based on Euler law and the microfluidic equivalent circuit was developed to understand the multiphysical phenomenon. Experimental data characterize the liquid flow stabilization performance of the flow stabilizer with multiple key parameters, such as the number and the size of microbubbles. The developed bubble-based hydraulic capacitor could minimize the flow pulses from syringe pumping by 75.3%. Furthermore, a portable system is demonstrated and compared with a commercial pressure-driven flow system. This study can enhance the understanding of the bubble-based hydraulic capacitors that would be beneficial in microfluidic systems where the precise and stable liquid flow is required.

## 1. Introduction

Microfluidics is the science and technology of systems that process small amounts of fluids, using channels with dimensions of tens to hundreds of micrometers [1]. It benefits many researchers in biomedical [2,3,4,5], cell sorting and culturing [6,7,8], disease diagnostic [9,10,11], and drug delivery [12,13] applications, among others. Fluidic actuation is one of the essential elements of a functional microfluidic system. Common fluidic actuation methods can be categorized as passive methods and active methods. Passive methods, including a capillary pump [14,15], thermal actuation [16], gravitational pumping, and so on, are dependent on fewer accessories, which allows them to be integrated with various devices. However, these passive methods usually fail to offer a well-controlled and consistent flow for practical applications.

Syringe pumps [17], pressure-driven pumps [18], and peristaltic pumps [19,20] are the most commonly used active pumping methods for microfluidic systems [21]. The syringe pump is the top option when the precisely controlled flow is required. However, the syringe pumps sometimes produce undesirable and insurmountable pressure pulses when working in low-flowrate conditions [22], due to the mechanical vibration from the stepper motor or poor maintenance. Therefore, the stability and accuracy of the syringe pump-driven flow are required to be improved for applications such as droplet and bubble generation [22,23].

Three categories of approaches were developed to provide stabilized flow with fewer pulses and fluctuations in syringe pump systems. One is to form several dampers serving as fluidic capacitance or cushions to minimal the fluidic fluctuations. The dampers are formed using either soft compliant material [24,25,26] or compressible air [21,27,28]. One is to deploy variation flow resistors to stabilize the unstable sample flow [29], and the other is to use magnetic [30] or electric actuators [31]. Much effort is devoted to attenuating syringe pump-induced fluctuations. However, there is comparatively little attention paid to theories to understand the flow stabilization process, as well as the manufacturability and integrability of the hydraulic stabilization device with an on-chip system.

**Figure 1:**
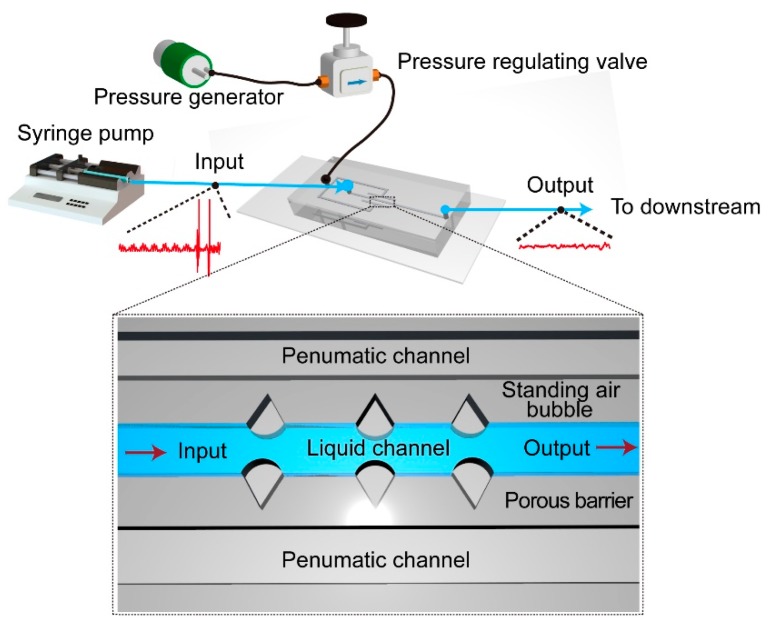
Schematic illustration of the bubble-based microfluidic stabilizer.

This paper studies the physical meaning of a novel hydraulic capacitor by using tunable micro-bubbles [32]. As shown in Figure 1, it consists of a group of crevices on the microfluidic channel wall, a pneumatic channel, and porous barriers isolating them. The micro air bubbles originate in and attach to the crevice structures, working as capacitance to attenuate pressure fluctuations from the upstream flow. The volume and the morphology of the microbubbles are well controlled by the pneumatic pressure. Theoretical models are developed to illustrate the physical principles of the microbubble-based microfluidic stabilizer, according to the Euler laws. Given the correlation between the Hagen–Poiseuille law and Ohm’s law, the well-known methods from electric circuit theory are applied to the microfluidic network, to further explain the mechanism behind the fluidic stabilization. It is further experimentally shown that, with the novel bubble-based fluid stabilization device, the flow fluctuation is maximally reduced to around 20.0%. The influence of the key parameters on the flow stabilization performance, such as microbubble morphology and quantity, are discussed via experimental data. Nevertheless, the microbubble stabilizer is compared with a pressure-driven system, which illustrates that it is capable of providing a stable and accurate liquid flow in a portable and cost-efficient manner. It also provides a novel method to understand compliance in a fluidic network.

## 2. Microbubble Formation and Control 

**Figure 2:**
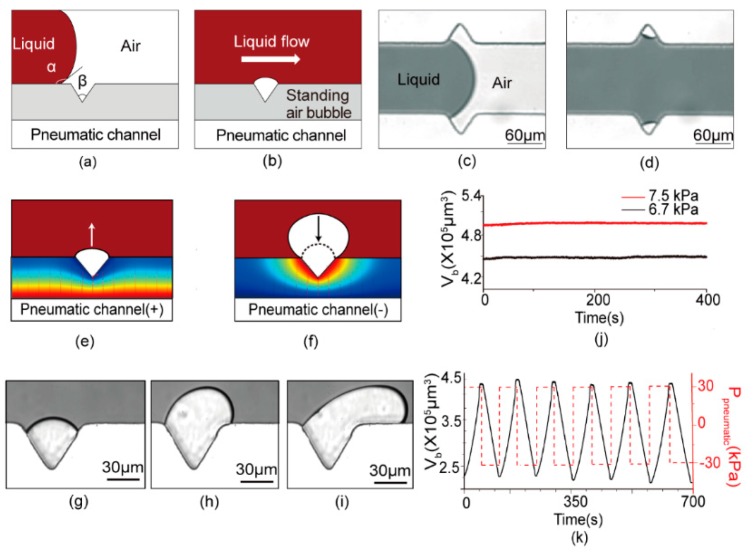
Generation and variation mechanism of the micro standing air bubble. (**a**,**b**) Schematic of a typical standing air bubble generation structure. (**c**,**d**) The microscopic observation of the bubble formation process. (**e**,**f**) Microfluidic standing air bubble (μSAB) volumetric variation principle, based on the ideal gas law, where the air diffusion leads to the μSAB volumetric enlargement/shrinkage under positive/negative pressure. (**g**–**i**) Top view of the bubble volume change process under a microscope. (**j**) The stability of the controllable standing air bubble under 5 μL/min flow rate at 7.5 kPa and 6.7 kPa. (**k**) Volumetric variation of μSAB under ±30 kPa cycles for more than 700 s.

Researchers found that small air bubbles could generate in the micro crevices on microchannel walls [33,34,35]. When certain conditions occur where the dynamic contact angle between the liquid and the channel walls (*α* in Figure 2a) is larger than the crevice inner angle (*β* in Figure 2a), there will be air left in the crevice structure after liquid flows by, since the liquid cannot fill all the space inside, as shown in Figure 2b.

When the microscale bubbles take shape in the crevices, they firmly attach to the channel during the operation under proper control. The top view of the bubble formation process under a microscope is shown in Figure 2c,d. PDMS (Polydimethylsiloxane) surfaces are sensitive to environmental conditions and handling protocols, becoming either hydrophilic or hydrophobic or varying between both states. However, this does not affect the formation process of the microbubble once the crevice angles are designed far away from the swing range of the advancing contact angle. The liquid we used was a mixture of pure water and blue inkjet printer ink, 5:1 in volume. It is commonly believed that the dynamic contact angle between normally made PDMS and the liquid we used is around 105° [36]. Therefore, the testing devices were designed to array 60° crevices on the fluidic channel walls to initiate and control these microbubbles.

The bubbles generated in the crevices can be tuned conveniently through the air/gas transfer between the fluid channel and the gas channel. PDMS is a porous material with selective permeability. It is permeable to air and some other gases while blocking the penetration of the liquid during gas diffusion. As Figure 2e illustrates, positive pressure exerted into the gas channel would lead to a pressure gradient directed to the bubble. Thus, the air is diffused from the gas channel into the bubble. The bubble begins to expand outward. On the contrary, the direction of diffusion is reversed due to negative pressure, causing the bubble to shrink inward, as shown in Figure 2f. Figure 2g–i shows the top view of the bubble variation process under the microscope. The volume calculation method is provided in the Supplementary Materials.

As found in Figure 2j, this bubble control strategy showed good stability on the bubble’s volume control. Under a stable flow condition, the bubble could keep its volume and size for a relatively long time. The bubble variation repeatability was also tested through an experiment of cyclical change of bubble volume for a relatively long time. The pressure in the pneumatic channel was periodically shifted between −30 kPa and 30 kPa, and the periodic variation of bubble volume was as shown in Figure 2k. The bubbles shrank or expanded at a nearly constant rate, in each pressure cycle. At the same time, the volume of the bubble remained stable within a range from 2.5 × 10^5^ μm^3^ to 4.5 × 10^5^ μm^3^. Furthermore, it can be found that both the volume variation rate and change illustrated a uniformity during each positive or negative pressure interval.

## 3. Theoretical Modeling of Flow Stabilization

### 3.1. Theoretical Model

The working principle of the bubble-based microfluidic stabilizer could be explained as outlined below. During an overflow, the air bubbles shrink in size, squeezed by the fluidic pressure. Thus, the pulse induced by the syringe pump can be weakened. As for underflow, the air bubbles begin to expand due to the pressure drop in the liquid channel, offsetting the decrease in flowrate. With these two statements, the fluctuation in flowing conditions can be eliminated, when fluid passes through the microfluidic stabilizer.

**Figure 3:**
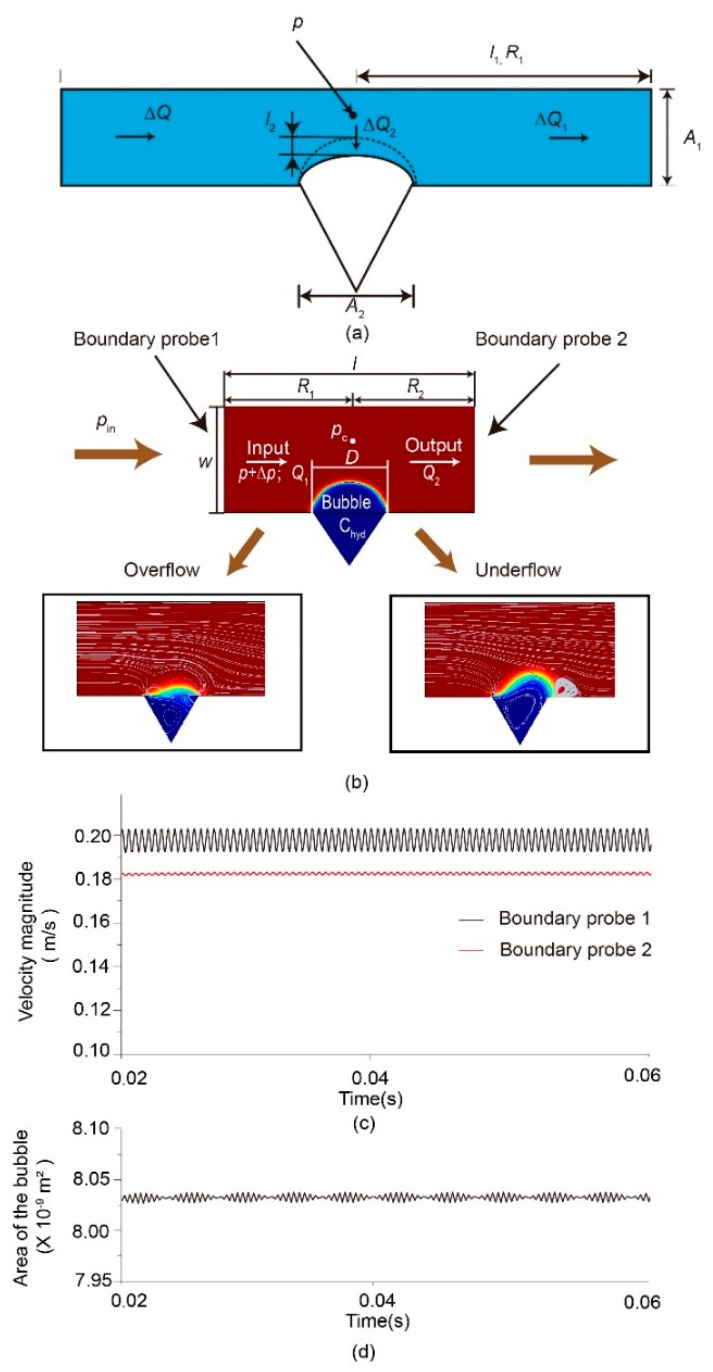
The mechanism of the μSAB flow stabilization effect: (**a**) the model to understand the effect of bubbles on the reduction of fluctuations; (**b**) the model for the simulation in COMSOL Multiphysics, and the storage and release process of the bubble-based damper. Here, part of the fluid will press the bubble for an overflow, and the fluid stored in the space of the bubble will be released back into the liquid channel for an underflow; (**c**) the flowrate velocity magnitude results of the simulation; (**d**) the bubble variation results of the simulation.

As shown in Figure 3a, in the steady state, the pressure in the conjunction of the bubble and the main channel is *p*, with the flowrate of *Q*_0_ in the main channel, while *ρ* is the density of the fluid. According to the Euler laws, the force can be derived as follows:

(1) $$\frac{dQ_{0}}{dt}\rho l_{1}=(p-Q_{0}R_{1})A_{1}=0,$$

where *A*_1_ is the cross-sectional area of the main channel, *l*_1_ is the length of the main channel, and *R*_1_ is the flow resistance of the main channel. The flowrate has a sharp increase in the upstream, resulting in a pressure increase Δ*p* in the conjunction. Suppose the increased flowrate is Δ*Q*. The force balance in the main channel can be derived as follows:

(2) $$\frac{d(Q_{0}+\Delta Q_{1})}{dt}\rho l_{1}=(p+\Delta p-Q_{0}R_{1}-\Delta Q_{1}R_{1})A_{1}=0,$$

(3) $$\frac{d\Delta Q_{1}}{dt}\rho l_{1}=(\Delta p-\Delta Q_{1}R_{1})A_{1},$$

where Δ*Q*_1_ is the increased flowrate in the downstream of the main channel. Through Laplace transformation, we obtain

(4) $$\Delta p(s)=\frac{\rho l_{1}\Delta Q_{1}}{A_{1}}s+\Delta Q_{1}R_{1}.$$

We considered the bubble as a mass-spring system. Considering that the temperature balance can be achieved in a very short time, and the mass diffusion process is quite short, the ideal gas law for the isothermal condition is given as *pV^γ^* = const(*γ* = 1). The pressure in the bubble is *p*_b_ = *p*_0_[*V*_0_/(*V*_0_ − Δ*V*)] = *p*_0_(1 − *l*_2_*A*_2_/*V*_0_). *p*_0_ is the initial pressure in the bubble, and *V*_0_ is the initial volume of the bubble. *A*_2_ is the cross-section area of the bubble crevice open area. *l*_2_ is the height of the bubble shrinkage, as shown in Figure 3a. For the mass-spring system, the stiffness coefficient is k = *p*_0_*A*_2_^2^/*V*_0_, *l*_2_ = ∫Δ*Q*_2_/*A*_2_ d*t*. The force balance between the bubble and fluid is given as

(5) $$\frac{d\Delta Q_{2}}{dt}\rho l_{2}=kl_{2}-\Delta pA_{2},$$

where Δ*Q*_2_ is the amount of flowrate to press the bubble. Through Laplace transformation, we obtain

(6) $$\Delta p(s)=\frac{p_{0}}{V_{0}}\frac{\Delta Q_{2}}{s}-\frac{\rho l_{2}Q_{2}}{A_{2}}s.$$

If we set *A*_1_ = 2*A*_2_ = 2*A*, the stabilization ratio λ is

(7) $$\lambda =\frac{\Delta Q_{1}}{\Delta Q_{1}+\Delta Q_{2}}=\frac{\frac{p_{0}A}{V_{0}}-2\rho l_{2}s^{2}}{\rho (l_{1}-2l_{2})s^{2}+2AR_{1}s+\frac{p_{0}A}{V_{0}}}.$$

Under the condition of slow flowrate in this paper, *l*_2_ is much lower than *l*_1_. It could be considered negligible. Therefore, the stabilization ratio *λ* can be rearranged as follows:

(8) $$\lambda =\frac{p_{0}}{\frac{\rho V_{0}}{A}s^{2}+2V_{0}R_{1}s+p_{0}}.$$

From the equation above, it is clear that the stabilizer can function as a fluidic stabilizer in a lab-on-chip system, which can minimize the fluctuation from the upstream. The stabilization effect is mainly affected by *V*_0_. Here, the volume of the bubble can be tuned easily using both the pressure in the pneumatic channel and the number of crevices on the channel. Thus, the device can be applied in different situations.

### 3.2. Finite Element Analysis

A numerical simulation was conducted in the COMSOL Multiphysics (Version 5.5, Stockholm, Sweden.) to further illustrate the principle of the introduced device for the flow regulation. The model established for the calculation is illustrated in Figure 3b. The width of the channel (*w*) was 150 μm, and the length of the channel (*l*) was 300 μm. The diameter (*D*) of the bubble was 60 μm, and the driven pressure *p*_in_ = 20 + 10sin(10^6^*t*) Pa. Two boundary probes were added to the inlet and outlet to obtain the velocity magnitude. Probe 1 stands for the inlet, while Probe 2 stands for the outlet. The two-phase flow was simulated using the level-set method.

When in the steady state, all the fluid flowing into the stabilizer will flow along the main channel to the outlet. Part of the fluid will press the bubble for an overflow, and the fluid stored in the space of the bubble will be released back into the liquid channel for an underflow, as shown in Figure 3b. Figure 3c demonstrates the flowrate velocity magnitude results at the inlet and outlet. Through the depicted curve, the input fluctuation is effectively surpassed. Figure 3d shows the variation of the bubble size. It is shown that the bubble size changes with the input flowrate, leading to a relatively stable flowrate at the output. Thus, the bubble-based fluidic stabilizer can function as a fluidic stabilizer similar to an electric filter. For more details, please refer to the Supplementary Materials Figure S1.

**Figure 4:**
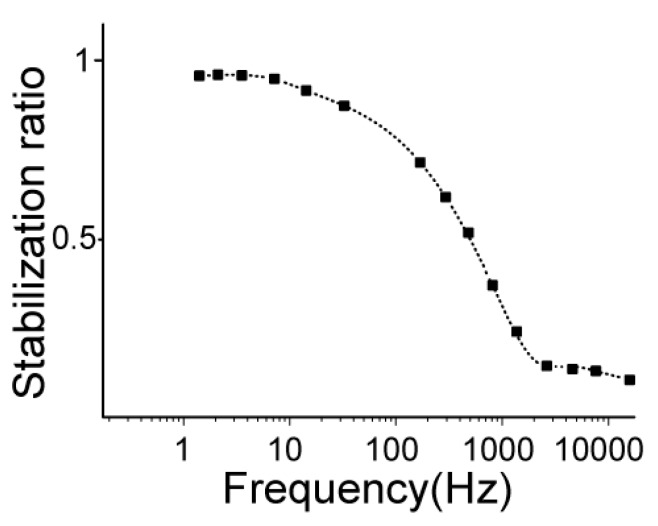
Frequency response of the bubble-based fluidic stabilizer obtained through simulation via COMSOL Multiphysics.

To further validate the working principle of the fluidic capacitor, another simulation in COMSOL Multiphysics was conducted using the model shown in Figure 3b. For more details, please refer to the Supplementary Materials Figure S2. According to Equation (7), the stabilization ratio λ is calculated by λ = Δ*Q*_1_/Δ*Q*. In this study, the flowrate profile was obtained through simulation. Figure 4 demonstrates that the fluctuation amplitude decreased by ~85% with the bubble-based fluidic stabilizer when the frequency exceeded 1000 Hz.

The relationship of the stabilization ration λ and fluctuation frequency is shown in Figure 4. The stabilization ratio decreased as frequency increased, which seemingly infers the filtering effect of the fluidic stabilizer.

## 4. Experiment and Verification

### 4.1. Chip Fabrication

To illustrate the flow-damping performance of the bubble-based stabilizer, several chips with bubble-generating crevices on the wall were firstly set up through the standard soft lithography process. Firstly, the pattern of the microchannel with the crevices inserted on the channel wall was designed through a computer-aided design (CAD) software program (AutoCAD 2019). The pattern created by the CAD was transformed into photomasks on transparency films by high-resolution printing. The master that contained the patterned relief structures on the surface was fabricated by lithography in photoresist SU-8 (Micro-Chem Corp, DURHAM, UK). The surface of the mold was salinized to make the surface hydrophobic. The PDMS base (Dow Corning Sylgard 184 Elastomer kit, Midland, MI, USA) and curing agent were mixed in a 10:1 mass ratio, stirring evenly. Subsequently, the bubbles in the mixture were passed through a vacuum drying oven. Hereafter, the mixture was poured on the mold and degassed and cured for two hours in an oven with a temperature of 60 °C. In this way, the microchannel with the crevice structures was formed at the same time. After curing, the PDMS stamp was separated from the master, then cut and punched. The PDMS and a glass slide were exposed to air plasma briefly and then bonded together, forming an entire microfluidic chip.

### 4.2. Experiment

**Figure 5:**
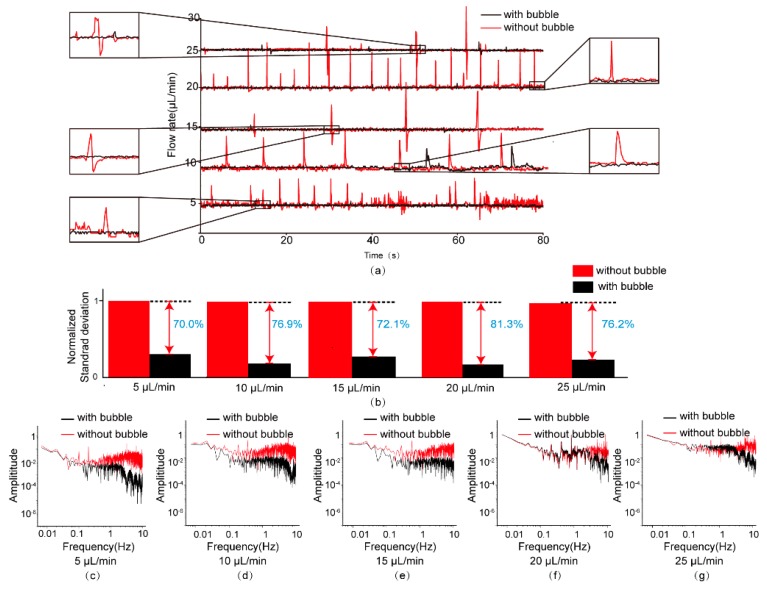
Characterization of flowrate fluctuation output using the syringe pumping set-up with/without bubbles. (**a**) The flowrate profiles produced by the stabilizer under different flowrates of 5 μL/min, 10 μL/min, 15 μL/min, 20 μL/min, and 25 μL/min. (**b**) Comparison of the normalized standard deviation of the syringe pumping flowrate with/without bubbles under different flowrates. (**c**–**g**) The spectrum analysis of the flowrate profiles produced by the stabilizer under different flowrates of 5 μL/min, 10 μL/min, 15 μL/min, 20 μL/min, and 25 μL/min.

As shown in Figure 1, the syringe pump was connected to the inlet of the micro stabilizer while the flow sensor was connected to the outlet for measurement of the flowrate. This set-up was constructed to measure the flowrate with/without the bubble existing. The immiscible fluid was introduced to the microchannel at 5 μL/min using the syringe pump. During the sample injection process, the air pressure exerted into the pneumatic channel was adjusted to generate bubbles for damping the flow fluctuation. Then, the pressure in the pneumatic channel was fixed to hold the bubble. The flowrate data were recorded, and they are represented with a black line in Figure 5. The fluidic flowrate data were obtained using the microfluidic flow sensor (Elveflow MFS 3 flow sensor 0 to +80 μL/min). Afterward, the air pressure was changed to a negative value to eliminate air bubbles. These flowrate data are represented by a red line (Figure 5a). As can be seen in Figure 5a, the damper could effectively surpass the pulse of the flow, with virtually no change in flowrate, thus achieving stabilization of the flow. At the same time, the normalized standard deviation of the flowrate in Figure 5b shows that the design of the bubble-based stabilizer could reduce the fluctuation of the flow and decrease the amplitude. Then, the flowrate determined by the syringe pump gradually increased from 5 μL/min to 25 μL/min. Through the FFT (Fast Fourier Transform) method, the flowrate profiles were processed as shown in Figure 5c–g. The record curves of the time constant under the positive effect of a well-damped system, in contrast to an undamped system, show that the set-up with the bubble-based micro-flow stabilizer achieved nearly the same flowrate output when absorbing the pulse caused by the stepper motor in a syringe pump, as shown in Figure 5a. For all set-ups with bubbles, magnitudes decreased as frequency increased; this seemingly indicates the low-pass filtering characteristics of the bubble-based fluidic stabilizer, as shown in Figure 5c–g.

**Figure 6:**
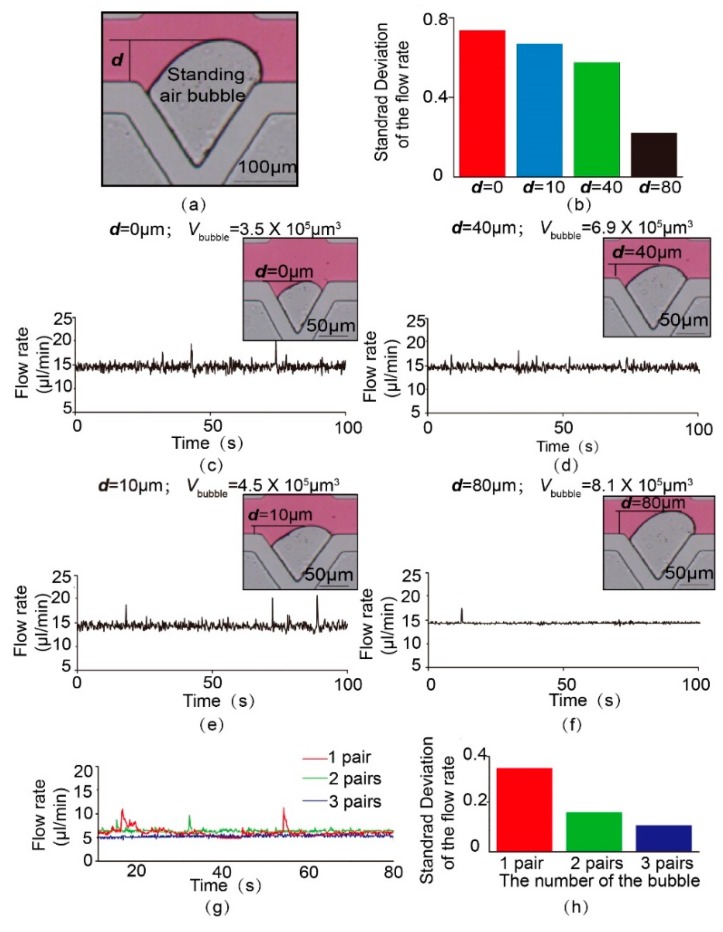
Output flowrates of bubble-based microfluidic stabilizer with different bubble sizes and numbers. (**a**) The illustration and experimental picture of the critical parameters affecting the flow stabilizing performance of this syringe stabilizer. (**b**) The standard deviation of the flowrate data with different sizes of the bubble. (**c**–**f**) Output flowrates of bubble-based microfluidic damper with different sizes of bubble (scale bar = 50 μm). (**g**) Output flowrates of bubble-based microfluidic stabilizer with different numbers of bubbles. (**h**) The standard deviation of the flowrate data with different numbers of bubbles.

According to previous knowledge, the size and the number of bubbles are the two most fundamental design parameters, since they significantly affect the flow-stabilizing performance of the stabilizer. This paper used the distance between the bubble top and the channel (*d*), together with the volume of the bubble (*V*), to characterize the size of the bubble (Figure 6a). The values of *d* and *V* can be simply tuned through the adjustment of the gas pressure in the pneumatic channel. This distance between the bubble top and channel (*d*) can be calibrated by the microscope’s vision software, while the volume of the bubble (*V*) can be later calculated using ImageJ. Only one bubble was used in this experiment. Figure 6c–f demonstrates the output flowrate of this bubble-based microfluidic stabilizer with different bubble sizes (*d* = 0 μm, *V* = 3.5 × 10^5^ μm^3^; *d* = 10 μm, *V* = 4.5 × 10^5^ μm^3^; *d* = 40 μm, *V* = 6.9 × 10^5^ μm^3^; *d* = 80 μm, *V* = 8.1 × 10^5^ μm^3^) under 15 μL/min flowrate. It was found from the experimental data that the output flowrate became more stable as *d* and *V* increased. As more bubbles invaded the flow channel, the flow became more stable. The column chart in Figure 6b also proves that larger bubbles could more effectively surpass the pulses under input flowrates including random fluctuations, which corroborates the conclusion of the theoretical model.

To explore the effect of the number of bubbles on the output flowrate, another three microfluidic on-chip stabilizers with one, two, and three pairs of crevices on the liquid channel wall were fabricated. The value of *d* in this experiment was set to 40 μm. Figure 6g illustrates the output flowrate of these three stabilization systems under the flowrate of 5 μL/min. Both the record curves of the flowrate in Figure 6g and the column chart in Figure 6h show that the increase in the number of bubbles could more effectively filter out the fluctuations and pulse, resulting in a more stable flow.

To illustrate the potential practicality of the system in this paper, we designed a portable microfluidic system integrated with a bubble-based fluidic stabilizer for smooth flowrate delivery in the downstream. The bubble-based fluidic stabilization was realized using cost-efficient and available tools. Generally, the easily assessible fluidic stabilization cost will not exceed $100 United States dollars (USD), while a precise pressure-driven pump can cost up to $1000 USD. For more details, please refer to the Supplementary Materials Figure S3.

## 5. Conclusion

In this paper, a cost-efficient and easy-to-fabricate fluidic stabilization was achieved via a controllable micro standing air bubble. The standing air bubble generation and variation mechanisms were briefly introduced. According to Euler’s equation, we illustrated the theoretical model behind the fluidic stabilization by considering the bubble as a mass-spring system. The relationship between the stabilization effect and several key parameters, such as the size of the bubble, was also demonstrated through the theoretical model. The flexible gas–liquid interface embedded in the microchannel functions as a hydraulic capacitor, compared to a circuit network. The microfluidic network can be regarded as an electrical filter.

The standard deviation of fluctuations was reduced to 24.3% by the microfluidic stabilizer. The experimental results illustrated that the size and the number of bubbles employed in the system had a direct and significant influence on the output flowrate. Furthermore, potential applications were introduced in this report. With a portable and low-cost damping set-up, a smooth flowrate was generated successfully. For more details, please refer to the Supplementary Materials Figure S3. This microfluidic stabilizer is suitable for different applications, such as hydrodynamic focusing, droplet generation, drug delivery, and particle synthesis.

## Supplementary Materials

The following are available online at https://www.mdpi.com/2072-666X/11/4/396/s1, Figure S1: Schematic and a circuit representation of microfluidic systems driven by a syringe pump, Figure S2: The result from the Comsol simulation, Figure S3: Application of the microfluidic stabilizer in the portable microfluidic system for stable flow-rate delivery.
