# Supplementary material for: Standing Air Bubble-Based Micro-Hydraulic Capacitors for Flow Stabilization in Syringe Pump-Driven Systems
Source: Micromachines (Basel). 2020 Apr 10;11(4):396. doi: 10.3390/mi11040396 (PMC7231304; doi:10.3390/mi11040396)
Supplement: Supplementary file 1 [file micromachines-11-00396-s001.pdf]

micromachines

Supplementary Materials: Standing Air Bubble-Based Micro-Hydraulic Capacitors for Flow Stabilization in Syringe Pump-Driven Systems

Yidi Zhou, Jixiao Liu, Junjia Yan, Tong Zhu, Shijie Guo, Songjing Li and Tiejun Li

1. Equivalent Circuit Model

In a microfluidic network, it can be found that a constant pressure drop will result in a constant flow rate. This result can be summarized in the Hagen-Poiseuille law [1]:

$$\Delta P = R_{\mathrm{hyd}} Q \tag{1}$$

The Hagen-Poiseuille law is completely analogous to Ohm's law. Thus, the proportionality factors R_{hyd} and C_{hyd} can be introduced as the hydraulic resistance and capacitance, respectively. The hydraulic resistance of a long straight channel with a rectangle cross-section shape can be given as:

$$R_{hyd} = \frac{12\eta L}{1-0.63(h/w)} \frac{1}{h^3 w} \tag{2}$$

where L is the length of the channel, μm; h is the height of the channel, μm, and w is the width of the channel, μm; η is the dynamic viscosity, mPa·s. Here η=1 mPa·s (water).

As the pressure increases by ΔP in a liquid the channel with a passive deformable part embedded in, the volume available to the liquid increases by ΔW. This process is very similar to the charging of the capacitor where an increase in voltage by ΔU increases the charge of the capacitor by $\Delta q = C\Delta U$. Therefore, the hydraulic capacitance is given by [2]

$$C_{\mathrm{hyd}} \equiv \frac{\mathrm{d}W}{\mathrm{d}P} \tag{3}$$

Thus, to make this fluidic stabilizer easier to understand, an equivalent circuit model was demonstrated in Figure S1. This report respectively corresponded the increased flowrate in the upstream ΔQ, the increased flow rate in the downstream of the main channel ΔQ_1 and the amount of flow rate to press the bubble ΔQ_2 to the increased current ΔI, ΔI_1, and ΔI_2. The hydraulic resistance of the upstream channel was corresponded to the R, and the hydraulic resistance of the downstream of the main channel was corresponded to the R_1, while, R_2 stands for the subsequent flow channel and component connected to this flow stabilizers. We treat it as a load during the calculation. In Figure S1, the transfer function is as follow:

$$H(j\omega) = \frac{\Delta I_1}{\Delta I} = \frac{\Delta I_1}{\Delta I_1 + \Delta I_2} \tag{4}$$

$$\frac{\Delta I_1}{\Delta I_2} = \frac{Z_{\mathrm{hyd}}}{R_1 + R_2} \tag{5}$$

Here, $Z_{\mathrm{hyd}} = 1/j\omega C_{\mathrm{hyd}}$, further rearrangement yields the following equations:

$$H(j\omega) = \frac{Z_{\mathrm{hyd}}}{R_1 + R_2 + Z_{\mathrm{hyd}}} = \frac{1}{(R_1 + R_2) C_{\mathrm{hyd}} j\omega + 1} \tag{6}$$

According to equation (3), the C_{hyd} of the bubble $C_{\mathrm{hyd}}^{\mathrm{bubble}}$ can be derived as:

$$C_{\text{hyd}}^{\text{bubble}} = \frac{V_0}{p_0}, \text{ with } [C_{\text{hyd}}] = \text{m}^3\cdot\text{Pa}^{-1} \tag{7}$$

where V_0 is the initial volume of the bubble, and p_0 is the initial pressure.

Therefore,

$$H(j\omega) = \frac{p_0}{(R_1 + R_2)V_0 j\omega + p_0} \tag{8}$$

According to the equation (8), the low pass filter attenuates fluctuations with frequencies higher than the cutoff frequency. The cutoff frequency of the low-pass filter is mainly decided by V0. The larger the size of the bubble is, the lower the cutoff frequency would be.

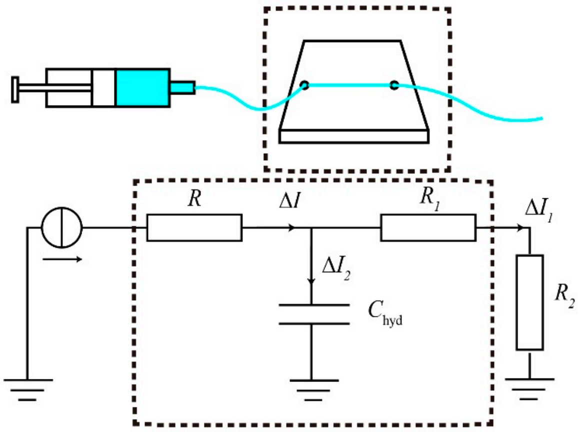

Figure S1. Schematic and a circuit representation of microfluidic systems driven by a syringe pump.

2. Bubble Volume Calculation

First, we import the experiment video into the ImageJ. In software, we use ImageJ's built-in algorithm to identify each frame of the video. That is, the image of each frame is converted into a grayscale image in the software. And the target area (bubble) and the background are marked according to the grayscale difference between the target and the background.

Then, with the help of the ImageJ's built-in algorithm, we can obtain the area data of the target area. This data is the cross-sectional area of the bubble from top view.

The volume of a bubble can be obtained by multiplying its area from top view by its height in the Z direction. Due to the shape and structure of the microchannel, we can consider the height of the channel as the height of the bubble. Thus, the volume of the bubble can be obtained by multiplying its area from top view by the height of the channel.

3. Additional Simulation

The driven pressure p_{in} = 20 +5sin(10^6t) +3sin(10^4t) +3sin(10^2t) Pa. And the result is shown in Figure 4. The input and output flow rate can be seen in the Figure S2a. The spectrum analysis of the results in Figure S2a is shown in Figure S2b. Both Figure S2a,b prove that the bubble-based fluidic stabilizer can function as a fluidic stabilizers similar to an electric filter.

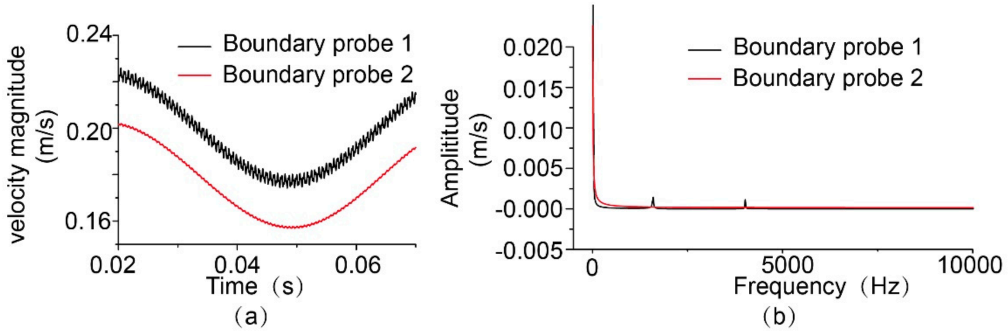

Figure S2. The result from the Comsol simulation. (**a**) the result of velocity magnitude; (**b**) the result of spectrum analysis**.**

4. Low-Cost and Precise Microfluidic Injection Application

To validate the practical application of the system in this paper we designed a portable microfluidic system integrated with a bubble-based fluidic stabilizer for the smooth flowrate delivery in the downstream. the bubble-based fluidic stabilization is realized using cost-efficiency and easy-accessible tools.

The schematic of the portable microfluidic system is shown in Figure S3a. The portable stabilizer is composed of a small diaphragm pump, a pressure regulating valve, and a microfluidic stabilizer chip. Here the small vacuum pump is used for bubble generation, and the pressure regulating valve for bubble controlling.

So far, the most widely used sample loading methods are pressure pumping and syringe pump. Syringe pump with fluidic stabilization set up can output relatively smooth flow, similar to the pressure pump as shown in Figure S3b. For syringe pump, the average flow rate in the device does not change due to the actual change in the device flow resistance. The amount of liquid injected is known for experiments. Thus, the syringe pump is the top option when the precisely controlled flow is required, compared with the pressure pumping method.

It is obviously illustrated in Figure S3b that with this bubble-based flow stabilizer, the pulse generated by the syringe pump can be effectively damped in a cost-efficiency and controllable method. Meanwhile, the flow rate output of this easy-accessible stabilizer with syringe pumping, and pressure pumping, has consistency in stability. The cost of a syringe pump with an easy-assessible fluidic stabilization set up, is relatively low, compared to a pressure pump. Generally, the easy-assessible fluidic stabilization's cost will not exceed 100 USD, while a precisely pressure driven pump can cost up to 1000 USD.

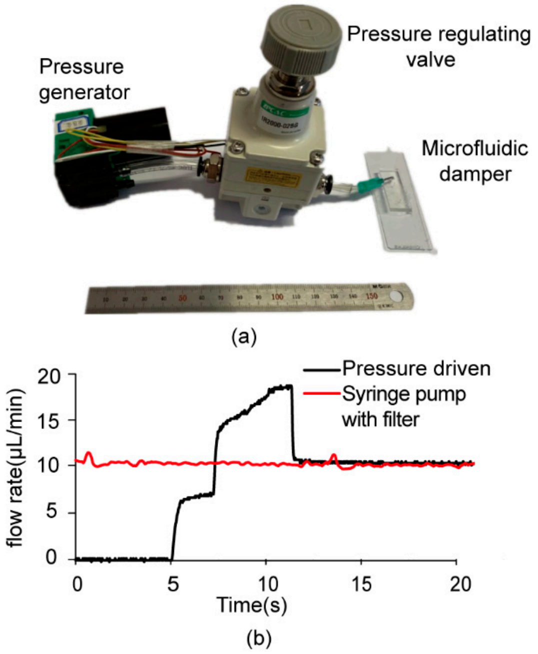

Figure S3. Application of the microfluidic stabilizer in the portable microfluidic system for stable flow-rate delivery. (**a**) The schematic of the portable microfluidic system composed of a small vacuum pump, a pressure regulating valve, and a microfluidic stabilization chip; (**b**) Comparisons of the flowrate profiles produced by syringe pumping with a damper and pressure-driven under the flowrate of 10 μL/min.

References.

1. Choi, S.; Lee, M.G. Microfluidic parallel circuit for measurement of hydraulic resistance. *Biomicrofluidics*, **2010**. *4*, 034110.
2. Laurell, T.; Lenshof, A. *Microscale acoustofluidics*; Royal Society of Chemistry: Cambridge, UK, 2004; pp. 18.
